# Tipping the Porter

**Published:** 1891-11-07

**Authors:** 


### PASSING TOPICS.

### "TIPPING THE PORTER."

EVER since Charles Dickens created and immortalised Bumble, and possibly long before that, it has been the fashion to hold, and not seldom to express, a very low opinion of the minor, to say nothing of the major, officials of parochial institutions. Of course, the habit of indulging in over-generalisation is faulty. Parish officers are not always monsters, and even beadles have their feelings. Similarly, hospital porters, who appear to come in for rather more than their fair share of contumely, may lose patience at last, and, as another of Dickens' characters pertinently exclaims under pathetic circumstances, "who can wonder?" The fact is that whenever there is a squabble or an allegation many persons proceed to judgment after hearing one side only. There is a great power in an *ex parte* statement, and to people content to decide upon mere assertion whatever appears in print is conclusive.

These reflections occur to us as we notice two letters about hospitals in contiguous columns of a morning paper, each dealing with an entirely different set of circumstances, but alike in the conclusion—that the porter was to blame. In the one case the statement is vouched for by the Chairman of the Committee. We are, therefore, precluded from supposing that it is simply an ingenious method of defence, and are forced to believe that the official gets his deserts. But in the other we have a sweeping charge made avowedly upon hearsay evidence. "Sir," writes a clergyman, "I should like to call the attention of the governors and directors of our London hospitals to the wretched custom that exists of 'tipping the porter.' I have heard many out-patients complain that they are kept waiting a long time, and the only way to prevent this is to put coins into the porter's pocket or to cross that important personage's palm." When the writer has made inquiry of medical men whether such a corrupt custom does really exist, the reply has *invariably* been, "Oh, yes, quite true; but you can't help it."

That a hospital porter, like other porters, is not averse from a "tip" is most likely true enough, but that the custom of tipping is so universal that the order in which patients are admitted is commonly regulated by these gratuities we take the liberty to doubt. Possibly certain offenders would be quite capable of reducing the exercise of their predatory instincts to a system if they dared, but we are disposed to think that hospital patients and their friends, as a class, are far too tenacious of their rights to let them slip in that way, and whatever particular patients may give and particular porters take, anything like wholesale jobbery could not fail of speedy detection in any well governed institution. It ought to be remembered, too, that the habit of offering tips is thoroughly ingrained in some people, and their favours are literally thrust upon the recipient without the asking. To expect a hospital porter at ten shillings a week to withstand this kind of temptation is asking almost too much, and if other patients who cannot "donate" are not prejudiced the harm is not great. Gratuities are, of course, forbidden. So they are on railway platforms, and porters and others are threatened with instant dismissal if found offending. But we never heard of a railway porter having been discharged for accepting a sixpence, though if, to put a parallel case to that set forth by the reverend gentleman, he took to demanding sixpences and delaying passengers who refused to give them, we think his career would be a short one. The gift or acceptance of a tip is not an immorality unless it sets seal to a conspiracy prejudicially affecting somebody else. In the latter case the giver is usually the worse of the two conspirators. The safeguard lies in the fact that the somebody else, especially if the term embodies a multitude, soon finds out and resents the injury. AGRICOLA.

